# Systematic discovery of drug action mechanisms by an integrated chemical genomics approach: identification of functional disparities between azacytidine and decitabine

**DOI:** 10.18632/oncotarget.8455

**Published:** 2016-03-29

**Authors:** Yao-Yu Hsieh, Tsui-Chin Huang, Hsiang-Ling Lo, Jyun-Yan Jhan, Shui-Tein Chen, Pei-Ming Yang

**Affiliations:** ^1^ PhD Program for Cancer Biology and Drug Discovery, College of Medical Science and Technology, Taipei Medical University and Academia Sinica, Taipei, Taiwan; ^2^ Division of Hematology and Oncology, Shuang Ho Hospital, Taipei Medical University, Taipei, Taiwan; ^3^ Graduate Institute of Cancer Biology and Drug Discovery, College of Medical Science and Technology, Taipei Medical University, Taipei, Taiwan; ^4^ Institute of Biological Chemistry, Academia Sinica, Taipei, Taiwan

**Keywords:** colorectal cancer, DNMT inhibitor, drug repurposing, polypharmacology, systems pharmacology

## Abstract

Polypharmacology (the ability of a drug to affect more than one molecular target) is considered a basic property of many therapeutic small molecules. Herein, we used a chemical genomics approach to systematically analyze polypharmacology by integrating several analytical tools, including the LINCS (Library of Integrated Cellular Signatures), STITCH (Search Tool for Interactions of Chemicals), and WebGestalt (WEB-based GEne SeT AnaLysis Toolkit). We applied this approach to identify functional disparities between two cytidine nucleoside analogs: azacytidine (AZA) and decitabine (DAC). AZA and DAC are structurally and mechanistically similar DNA-hypomethylating agents. However, their metabolism and destinations in cells are distinct. Due to their differential incorporation into RNA or DNA, functional disparities between AZA and DAC are expected. Indeed, different cytotoxicities of AZA and DAC toward human colorectal cancer cell lines were observed, in which cells were more sensitive to AZA. Based on a polypharmacological analysis, we found that AZA transiently blocked protein synthesis and induced an acute apoptotic response that was antagonized by concurrently induced cytoprotective autophagy. In contrast, DAC caused cell cycle arrest at the G_2_/M phase associated with p53 induction. Therefore, our study discriminated functional disparities between AZA and DAC, and also demonstrated the value of this chemical genomics approach that can be applied to discover novel drug action mechanisms.

## INTRODUCTION

Many therapeutic drugs generally exhibit actions on more than one molecular target, which is a phenomenon known as polypharmacology [1]. Predicting the polypharmacology of clinically used drugs or compounds that failed during development is highly useful for finding new indications (drug repurposing or repositioning) and discovering novel drug action mechanisms to improve their therapeutic efficacies [2]. Increasingly, large-scale databases are being established to explain how drugs induce changes in gene and protein expressions in human cells and affect their phenotypes on a global scale [3]. Utilization and integration of these resources can build a systematic view of a drug's actions. For example, our previous study successfully identified novel action mechanisms of the Chinese herbal medicine, berberine, using a gene expression signature-based approach [4]. This approach integrates the Connectivity MAP (CMAP) that collects gene-expression profiles from cultured human cells treated with small molecules [5], and the Search Tool for Interactions of Chemicals (STITCH) that explores known and predicted interactions of chemicals and proteins by evidence derived from experiments, databases, and the literatures [6]. More and more biomedical databases and analytical tools have been developed in recent years, which provide easy and open access to masses of accumulated data [3]. Integration of these resources will be highly useful in the field of polypharmacology.

DNA methylation is one of the most widely studied epigenetic changes, which is catalyzed by DNA methyltransferases (DNMTs). Two DNMT inhibitors, 5-azacytidine (azacitidine, AZA) and 2′-deoxy-5-azacytidine (decitabine, DAC) were approved by the US Food and Drug Administration for treating myelodysplastic syndrome and other leukemias [7]. Both drugs are cytidine analogs that are incorporated into DNA to bind and inhibit DNMTs, thus preventing maintenance of the methylation status. Unlike DAC, which is directly incorporated into DNA, AZA is primarily incorporated into RNA. Only when its diphosphate form is reduced to deoxy-diphosphates by ribonucleoside reductase can AZA then be incorporated into DNA to deplete DNMTs [8–10]. Actually, AZA was reported to more often be incorporated into RNA than DNA [9, 10]. Therefore, AZA may possess RNA-dependent effects. Indeed, ribonucleotide reductase M2 (RRM2), a subunit of ribonucleotide reductase, was identified as a novel molecular target of AZA in acute myeloid leukemia [11]. The inhibition of RRM2 expression by AZA involves its direct RNA incorporation and attenuated RRM2 messenger (m)RNA stability [11]. Thus, AZA and DAC should be viewed as distinct types of DNMT inhibitors, and a greater understanding of their action mechanisms will provide further clinical benefits.

In this study, we used an integrated chemical genomics approach to investigate functional disparities between AZA and DAC. This approach integrated the Library of Integrated Cellular Signatures (LINCS) [12] which is the next generation of CMAP, together with two analytical tools, the STITCH and the WEB-based GEne SeT AnaLysis Toolkit (WebGestalt). Polypharmacological analyses of AZA and DAC displayed functional disparities that were further demonstrated by proof-of-concept studies which showed that AZA transiently blocked protein synthesis and reduced protein stability, while DAC induced cell cycle arrest at the G_2_/M phase that was associated with p53 expression. Further investigation showed that AZA simultaneously induced an acute apoptotic response and cytoprotective autophagy in a mutually exclusive manner. Our results provide an example of applying several existing bioinformatics tools for systematic discovery of polypharmacology.

## RESULTS

### Utilization of an integrated chemical genomics workflow for polypharmacology

Polypharmacology, described as the binding of a drug to more than one target, can lead to multiple outcomes. Unfortunately, methods for exploring the polypharmacology of drugs are still lacking. CMAP, a chemical genomics database that collects gene-expression profiles from cultured human cells treated with small molecules, can be used to find connections among small molecules that share common action mechanisms [5]. The LINCS is the next generation of CMAP, which generates gene expression signatures including pharmacological or genetic perturbations applied to mostly cancer cell lines using the L1000 platform [13]. The L1000 assay is an mRNA expression profiling technique based on reduced representation of the genome whereby 1000 carefully selected transcripts are monitored, and from which the remainder of the transcriptome can be computationally inferred [13]. Compared to CMAP, the LINCS contains gene expression profiles of small molecules and also those of genetic constructs for knocking-down genes (short hairpin (sh)RNA) or overexpressing genes (complementary (c)DNA). The LINCS has greater numbers of gene expression signatures (perturbagens) and cell lines than CMAP, thus providing more-reliable predictions. In addition, the LINCS can be more easily accessed through a web-based interface at http://www.lincscloud.org/. Therefore, we proposed that the LINCS provides an opportunity for a comprehensive systematic analysis of polypharmacology.

**Figure 1:**
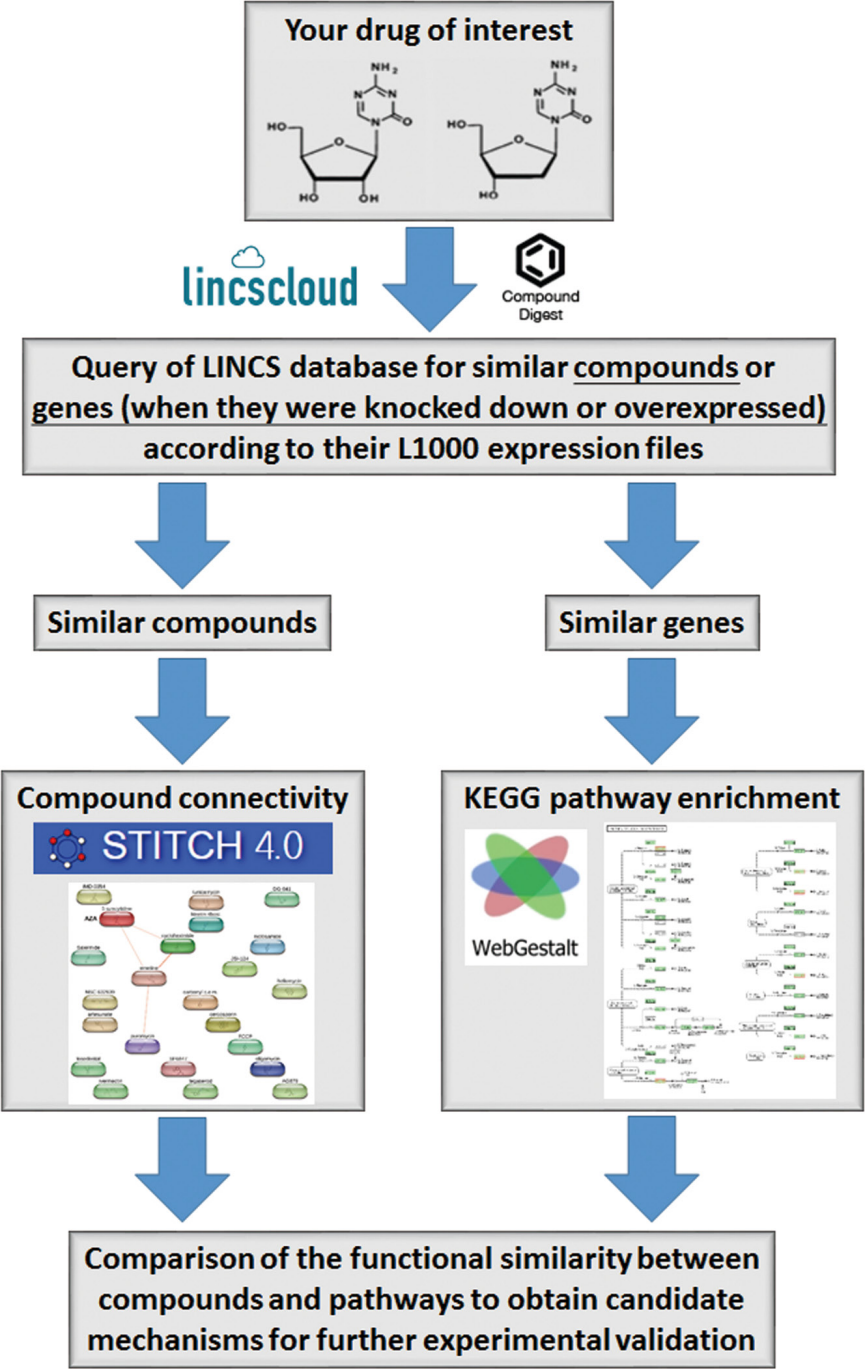
Workflow for the integrated chemical genomics approach

Herein, we describe the workflow of a polypharmacology analysis that integrated the LINCS with other online bioinformatics tools (Figure 1). First, we queried the LINCS database (http://www.lincscloud.org/) for “your drug of interest” using the “Compound Digest” algorithm. We obtained output results for “Compound Connections”, “Consensus Knockdown Connections”, and “Overexpression Connections”. The “Compound Connections” section contained a number of compounds similar to “your drug of interest”. The “Consensus Knockdown Connections” and “Overexpression Connections” sections indicated that the outcomes of knockdown or overexpression of genes were similar to the effects of “your drug of interest”. Second, compounds with strongly positive connectivity scores in “Compound Connections” were further filtered using the STITCH database (http://stitch.embl.de/) to explore their chemical-chemical connectivities [6]. Then, compounds directly connected to “your drug of interest” were obtained. Third, gene profiles of “Consensus Knockdown Connections” and “Overexpression Connections” were analyzed using WebGestalt (http://bioinfo.vanderbilt.edu/webgestalt/) [14] to enrich Kyoto Encyclopedia of Genes and Genomes (KEGG) pathways. Therefore, the polypharmacology of “your drug of interest” was obtained from results of the STITCH and WebGestalt analyses. By comparing results of the STITCH and WebGestalt analyses, we obtained possible targets or functions regulated by “your drug of interest” for further validation.

### Differential effects of AZA and DAC on human colorectal cancer (CRC) cells

**Figure 2:**
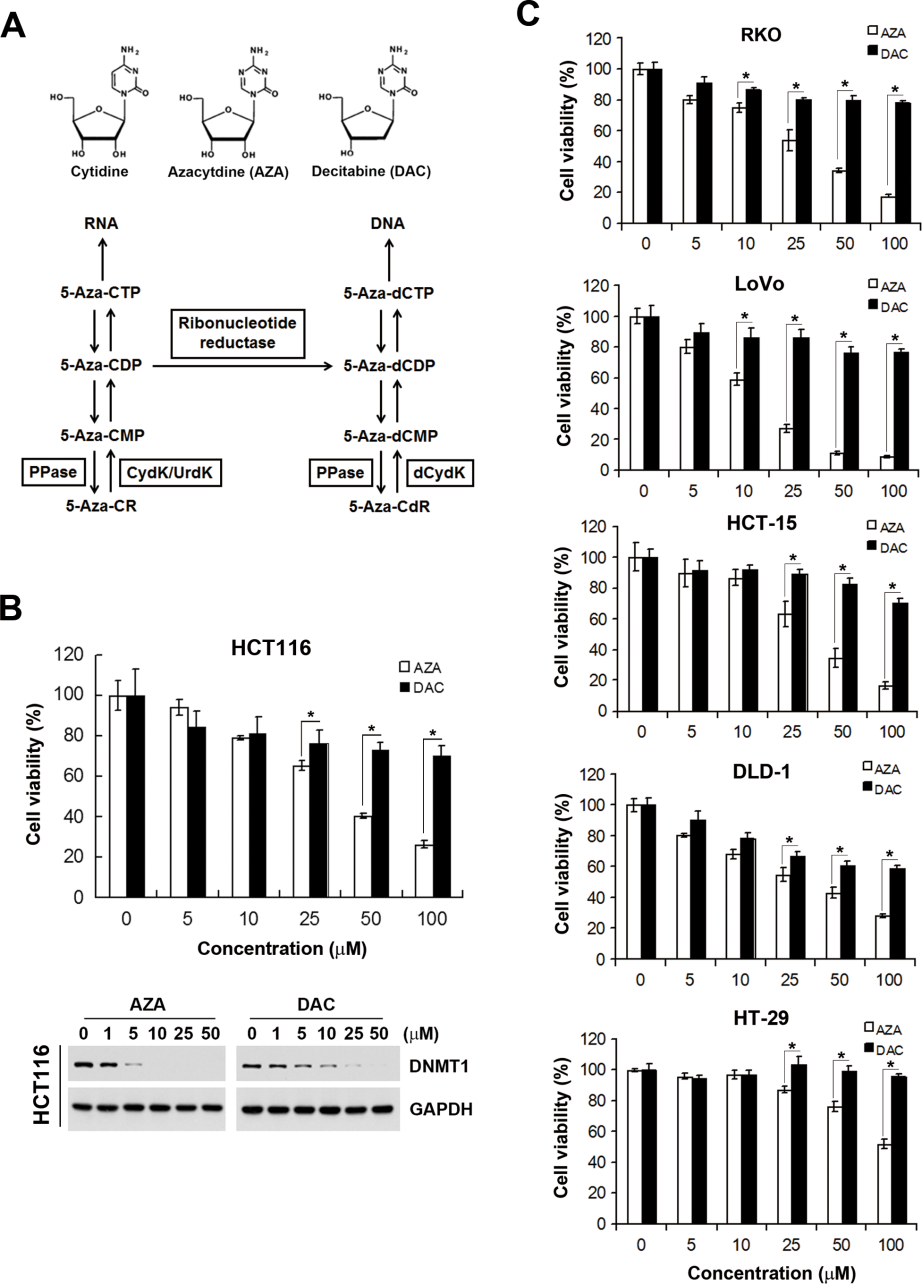
Different effects of azacytidine (AZA) and decitabine (DAC) on the cell viability of human colorectal cancer cells (**A**) Chemical structures of cytidine, AZA, and DAC, and the metabolic pathways of AZA (5-Aza-CR) and DAC (5-Aza-CdR). MP, DP, and TP, mono-, di-, and triphosphate, respectively; PPase, phosphatase; UrdK/CydK, uridine/cytidine kinase; dCydk, deoxycytidine kinase. (**B**) HCT116 cells were treated with different doses of AZA or DAC for 24 and 72 h. The cell viability at 72 h was analyzed by an MTT assay (upper part). Whole-cell lysates at 24 h were subjected to a Western blot analysis using antibodies against DNMT1 or GAPDH (lower part). (**C**) RKO, LoVo, HCT-15, DLD-1, and HT-29 cells were treated with different doses of AZA or DAC for 72 h, and the cell viability was analyzed by an MTT assay.

To demonstrate the feasibility of our chemical genomics strategy, the polypharmacological analyses of two DNMT inhibitors, AZA and DAC, were performed and used as examples. Despite the similarity of AZA and DAC, which both closely resemble the structure of cytidine, their metabolism and destinations in cells were distinct (Figure 2A). DAC is phosphorylated by deoxycytidine kinase into triphosphate, which can be incorporated into newly synthesized DNA. In contrast, AZA is primarily phosphorylated by uridine-cytidine kinase into triphosphates, which are ultimately incorporated into RNA. However, the diphosphate forms of AZA can also be reduced by ribonucleoside reductase into deoxy-diphosphates, which can be incorporated into DNA. It was reported that incorporation of AZA into RNA is greater than that into DNA [9, 10]. Therefore, AZA may possess RNA-dependent effects. Indeed, differential responses of AZA and DAC toward various types of cancers were observed [15–19]. In this study, we also observed that human CRC HCT116 cells were more sensitive to AZA than to DAC according to an MTT cell viability assay (Figure 2B, upper part; IC_50_ values shown in Table 1). To investigate whether the differential effects of AZA and DAC result from their ability to degrade DNMTs, a Western blot analysis of DNMT1 was performed. As shown in Figure 2B (lower part), DNMT1 was depleted in response to both AZA and DAC, suggesting that other mechanisms in addition to DNMT1 inhibition are involved in their differential effects. To investigate whether this phenomenon is common in human CRC cells, several cell lines (RKO, LoVo, HCT-15, DLD-1, and HT-29) were treated with AZA or DAC for 3 days, and then cell viabilities were analyzed by an MTT assay. Consistently, these cell lines were more sensitive to AZA treatment (Figure 2C). Therefore, we propose that AZA and DAC are suitable examples to evaluate whether our approach can discriminate their functional disparities.

**Table 1:** 50% inhibitory concentration (IC_50_) values of azacytidine (AZA) and decitabine (DAC) against colorectal carcinoma (CRC) cell lines

	IC_50_ of AZA (μM)	IC_50_ of DAC (μM)
HCT116	38.2	ND^a^
RKO	28.7	ND
LoVo	12.5	ND
HCT-15	34.4	ND
DLD-1	32.8	ND
HT-29	104.3	ND

a ND, not determined due to the low cytotoxicity of DAC.

### Chemical genomics analysis reveals functional disparities between AZA and DAC

**Figure 3:**
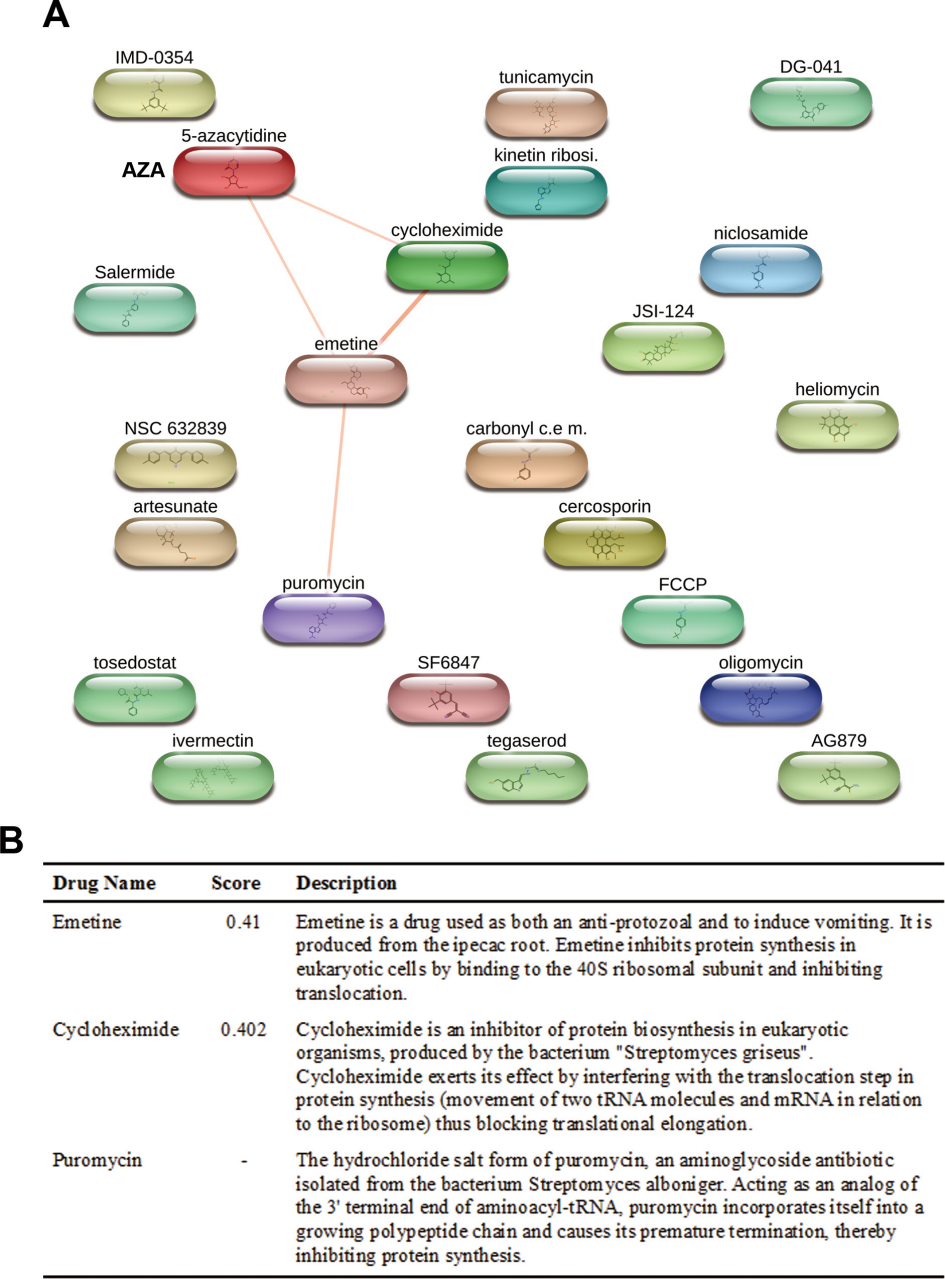
Prediction of compounds similar to azacytidine (AZA) by STITCH Chemical connectivity analysis was performed using the STITCH database as described in “Materials and Methods”. In (**A**), a diagram shows compounds connected to AZA. In (**B**), a table shows connectivity scores of compounds linked to AZA, and their functional descriptions.

Herein, we analyzed the polypharmacology of AZA and DAC by our chemical genomics workflow (Figure 1). First, we queried the LINCS database for AZA (input “azacitidine”) or DAC (input “decitabine”) using the “Compound Digest” algorithm. We obtained the output results of “Compound Connections”, “Consensus Knockdown Connections”, and “Overexpression Connections”. The top 100 compounds with positive scores in “Compound Connections” are shown in Supplementary Table S1, representing the most similar drugs to AZA or DAC. These drugs were further queried using the STITCH database to explore their chemical-chemical connectivities [6]. As shown in Figure 3A, the STITCH analysis indicated that AZA is directly connected to emetine and cycloheximide (CHX), and indirectly to puromycin. These drugs belong to protein synthesis inhibitors (Figure 3B). In contrast, DAC did not have direct connections to other compounds (data not shown). Therefore, these results suggest that AZA and DAC indeed show functional disparities, and AZA might interfere with protein synthesis.

Output results of “Consensus Knockdown Connections” and “Overexpression Connections” indicate that knockdown and overexpression of genes with strong positive scores have similar gene expression signatures to AZA or DAC. Analyzing these genes may reveal action mechanisms of AZA and DAC. Therefore, enrichment of KEGG pathways was evaluated using the WebGestalt [14]. Due to limited genes with a score of > 60 in “Overexpression Connections” gene sets of AZA and DAC (Supplementary Table S2), only “Consensus Knockdown Connections” gene sets were analyzed. As shown in Table 2, “aminoacyl-tRNA biosynthesis” and “metabolic pathways” were enriched in the “Consensus Knockdown Connections” gene set of AZA. While in the “Consensus Knockdown Connections” gene set of DAC, numerous pathways were enriched, including “pathways in cancer”, “cell cycle”, “p53 signaling pathway”, “metabolic pathways”, “T cell receptor signaling pathway”, “epithelial cell signaling in *Helicobacter pylori* infection”, “neurotrophin signaling pathway”, “Chagas disease (American trypanosomiasis)”, and “osteoclast differentiation”. Consistent with results of the STITCH analysis, AZA and DAC showed functional disparities in regulating biological pathways. Results of the STITCH and WebGestalt analyses were comparable because aminoacyl-tRNAs are substrates for protein translation [14], and AZA might disrupt aminoacyl-tRNA biosynthesis and inhibit protein synthesis. Although DAC did not show direct connections to other compounds in the STITCH analysis, a pathway enrichment analysis indicated that DAC might affect cell cycle progression and the p53 signaling pathway (Table 2). In addition, the most similar compound to DAC was danusertib (Supplementary Table S1) which was formerly known as PHA-739358 and acts as a pan-Aurora kinase inhibitor [20]. Aurora kinases consist of a family of serine/threonine kinases that play important roles in cell cycle progression, particularly during the G_2_ and M phases [21]. Danusertib was found to arrest cancer cells at the G_2_/M phase involving the p53 signal pathway [22]. Therefore, we propose that DAC may alter the phenotype of cells similar to that by danusertib.

**Table 2:** Gene set enrichment analysis (GSEA) for KEGG pathways enriched (*p* < 0.01) in consensus knockdown genes connected to azacytidine (AZA) or decitabine (DAC)

	Pathways	Genes	No. of genes in pathway	No. of differentially expressed pathway genes (% of total)	*p* value
**AZA**	Aminoacyl-tRNA biosynthesis	EPRS, MARS, LARS, WARS2, IARS2	63	5 (7.94%)	7.66e–07
	Metabolic pathways	CAT, SRM, EPRS, CYP27B1, ALDH3B1, TSTA3, NME4, CBR3, ALDH18A1, ARG1, PMM2, SDHA, FAH	1130	13 (0.53%)	2.22e–06
**DAC**	Pathways in cancer	JUN, RB1, PIAS1, IGF1R, PPARG, CDK4, RET, PIAS2, HDAC1, SMAD4, FADD, FAS	326	12 (3.68%)	3.53e–11
	Cell cycle	RB1, CDK4, PRKDC, ATM, HDAC1, CHEK1, CHEK2, CCNB1, SMAD4	124	9 (7.26%)	3.53e–11
	p53 signaling pathway	ATM, CHEK1, CHEK2, CCNB1, CDK4, FAS, THBS1	68	7 (10.29%)	4.50e–10
	Metabolic pathways	SUCLA2, PDHA1, ENPP1, GRHPR, GBGT1, AGPAT2, PGK1, PAH, PAICS, CDO1, ACLY, AK4, UQCRC1, OGDH, ACACA	1130	15 (1.33%)	4.48e–08
	T cell receptor signaling pathway	JUN, MAPK13, LCK, MAPK14, CDK4, MALT1	108	6 (5.56%)	2.47e–07
	Epithelial cell signaling in Helicobacter pylori infection	JUN, MAPK13, CSK, MAPK14, LYN	68	5 (7.35%)	5.04e–07
	Neurotrophin signaling pathway	IRS1, JUN, MAPK13, CSK, MAPK14, PRKCD	127	6 (4.72%)	5.04e–07
	Chagas disease (American trypanosomiasis)	JUN, MAPK13, MAPK14, FADD, FAS	104	5 (4.81%)	3.66e–06
	Osteoclast differentiation	JUN, MAPK13, LCK, PPARG, MAPK14	128	5 (3.91%)	8.98e–06

Consensus knockdown genes with a rank of < 100 and/or a Score-best6 of > 60 were analyzed by a web-based enrichment analytical tool WebGestalt (http://bioinfo.vanderbilt.edu/webgestalt/).

### Proof-of-concept studies for functional disparities between AZA and DAC

**Figure 4:**
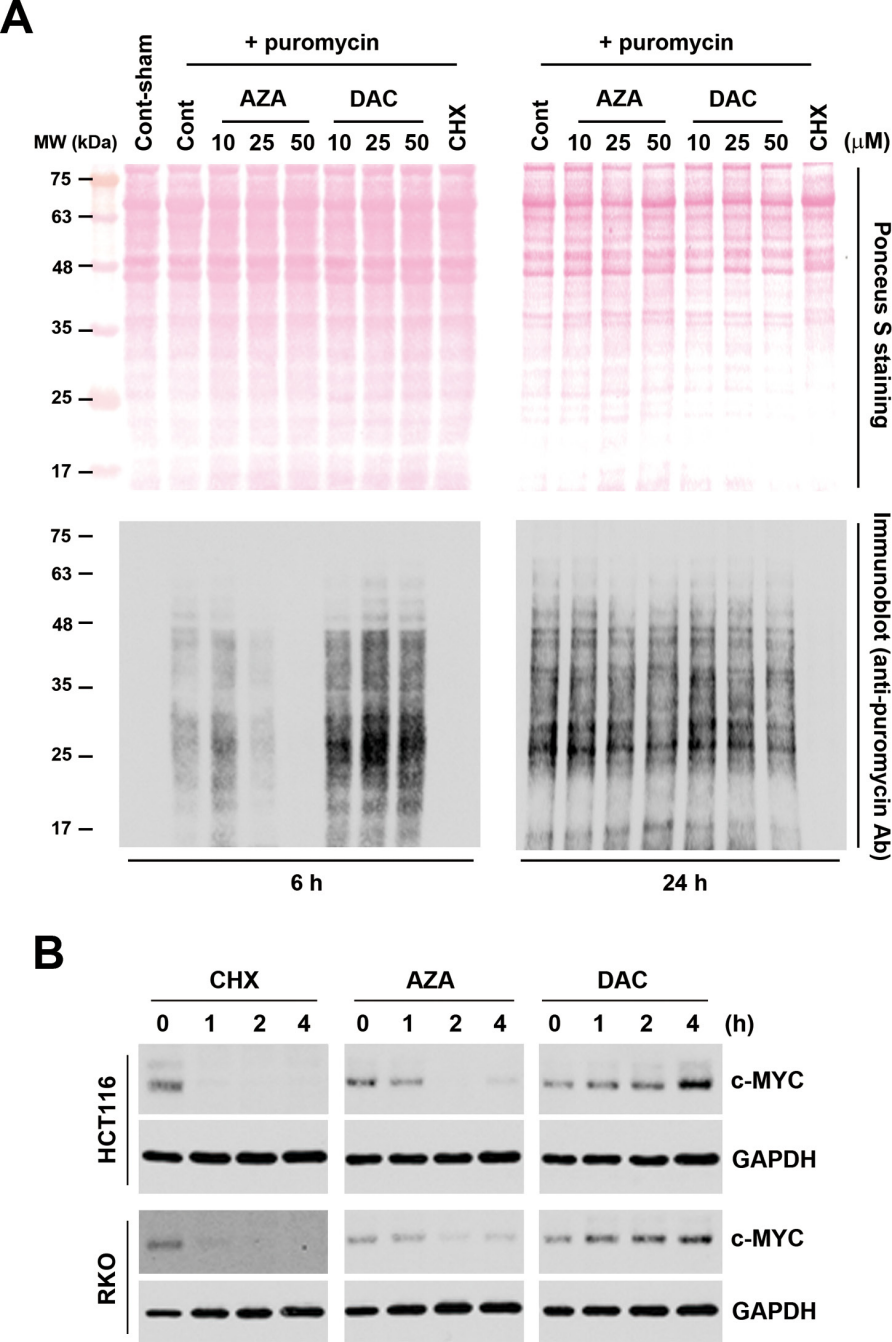
Effects of azacytidine (AZA) and decitabine (DAC) on protein synthesis and stability (**A**) HCT116 cells were treated with 5 μg/mL cycloheximide (CHX), or different doses of AZA or DAC for 6 and 24 h, and protein synthesis was examined by a puromycin-incorporation assay as described in “Materials and Methods”. Upper part: ponceau S staining of nitrocellulose membranes. Lower part: Western blots of puromycin. (**B**) HCT116 and RKO cells were treated with 5 μg/mL CHX, 50 μM AZA, or 50 μM DAC for the indicated time intervals, and whole-cell lysates were subjected to a Western blot analysis using antibodies against c-MYC or GAPDH.

Based on results of the chemical genomics analyses, we hypothesized that AZA disrupts aminoacyl-tRNA biosynthesis and then inhibits protein synthesis, while DAC perturbs cell cycle progression that may involve the p53 signaling pathway. To demonstrate this hypothesis, we first analyzed protein synthesis by a puromycin-incorporation assay [23]. As a Tyr-tRNA mimetic, puromycin enters the ribosome A site and terminates translation through its covalent incorporation into the C terminus of nascent polypeptide chains [24]. Puromycin-incorporated neosynthesized proteins can be detected by an anti-puromycin antibody [23]. To investigate the effects of AZA and DAC on protein synthesis, HCT116 cells were treated with AZA or DAC for 6 and 24 h, or with CHX, a protein synthesis inhibitor, which was used as a positive control. Puromycin was added to cells 30 min before cells were harvested. Whole-cell lysates were separated using SDS-PAGE and transferred to a membrane. Proteins were visualized using ponceau S staining (Figure 4A, upper part). After hybridization with an anti-puromycin antibody, we found that AZA, like CHX, inhibited puromycin incorporation at 6 h; however, DAC enhanced puromycin incorporation at the same time (Figure 4A, lower part). Interestingly, the effects of AZA and DAC had recovered at 24 h, indicating that alterations of protein synthesis by AZA and DAC were transient. Because perturbation of protein synthesis will affect protein stability, we further examined the effects of AZA, DAC, and CHX on protein stability by treating HCT116 and RKO cells with these drugs for 1∼4 h. We chose the c-MYC protein as an indicator because of its short half-life, usually 20∼30 min [25]. As shown in Figure 4B, both AZA and CHX reduced the protein stability of c-MYC in HCT116 and RKO cells. In contrast, DAC increased the stability of c-MYC.

**Figure 5:**
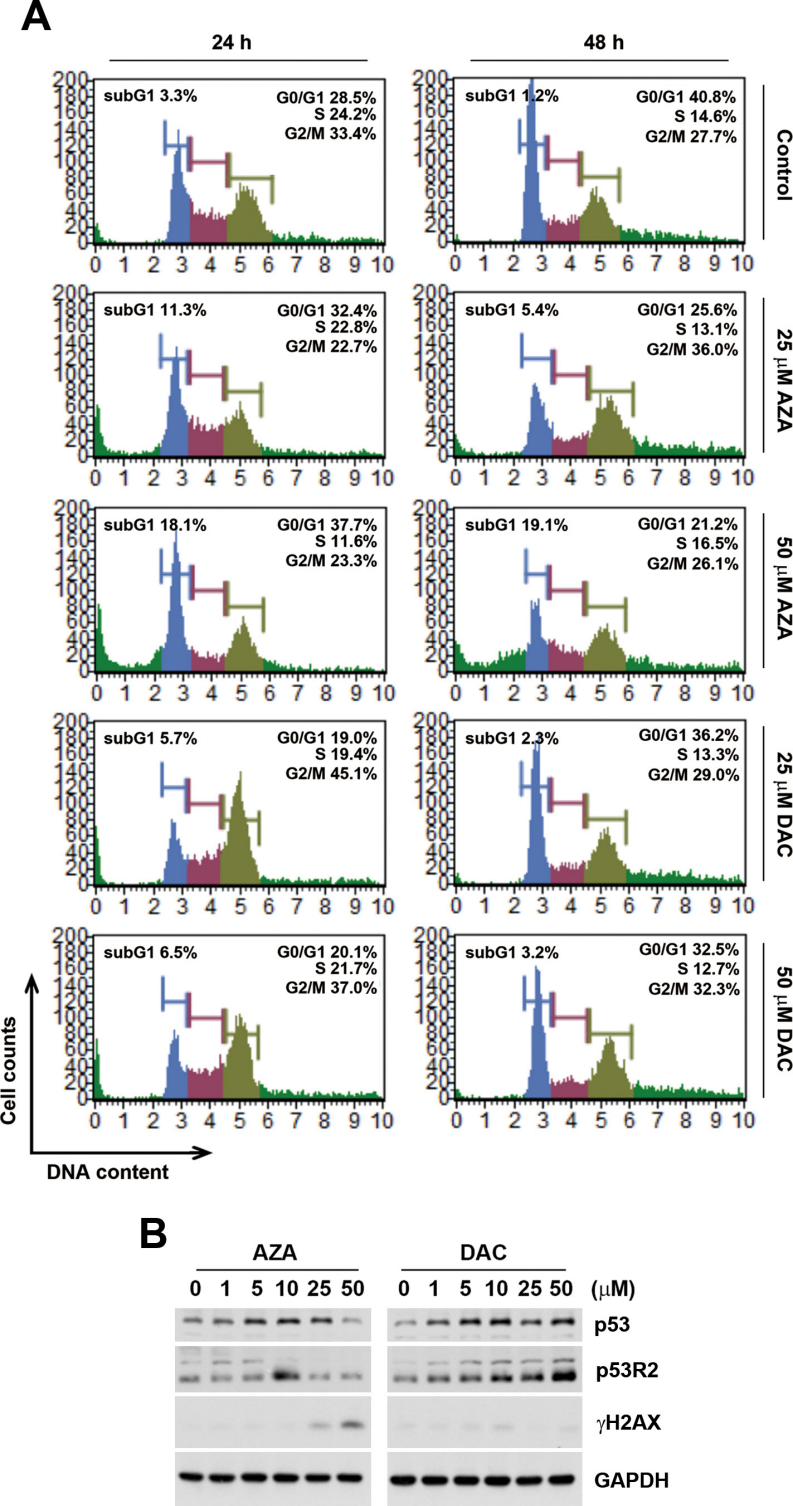
Effects of azacytidine (AZA) and decitabine (DAC) on the cell cycle and p53 expression (**A**) HCT116 cells were treated the different doses of AZA or DAC for 24 and 48 h, and the cell cycle was analyzed by flow cytometry as described in “Materials and Methods”. (**B**) HCT116 cells were treated the different doses of AZA or DAC for 24 h, and whole-cell lysates were subjected to a Western blot analysis using antibodies against p53, p53R2, γH2AX, or GAPDH.

We also examined the effects of AZA and DAC on cell cycle progression and the p53 signaling pathway according to the above prediction. To investigate whether AZA or DAC affected cell cycle progression, HCT116 cells were treated with 25 and 50 μM AZA or DAC for 24 and 48 h, and then cell cycle distributions were analyzed by flow cytometry. Consistent with results of pathway enrichment, DAC induced G_2_/M arrest at 24 h, which had been relieved by 48 h (Figure 5A). In contrast, AZA increased the subG_1_ population at both 24 and 48 h, suggesting the induction of apoptosis (Figure 5A). To investigate the effects of AZA and DAC on the p53 signaling pathway, protein expressions of p53 and p53R2, a p53 target gene that causes G_2_/M arrest and is directly involved in the p53 checkpoint for DNA repair [26], were analyzed by a Western blot analysis. As shown in Figure 5B, DAC induced greater expressions of p53 and p53R2 compared to AZA. In addition, AZA, but not DAC, induced DNA damage as indicated by the phosphorylation of H2AX (γH2AX), indicating that DAC-induced p53R2 might be responsible for the repair of DNA damage. Taken together, these proof-of-concept studies confirmed our chemical genomics approach.

### AZA induces an acute apoptotic response that is antagonized by concurrent autophagy

**Figure 6:**
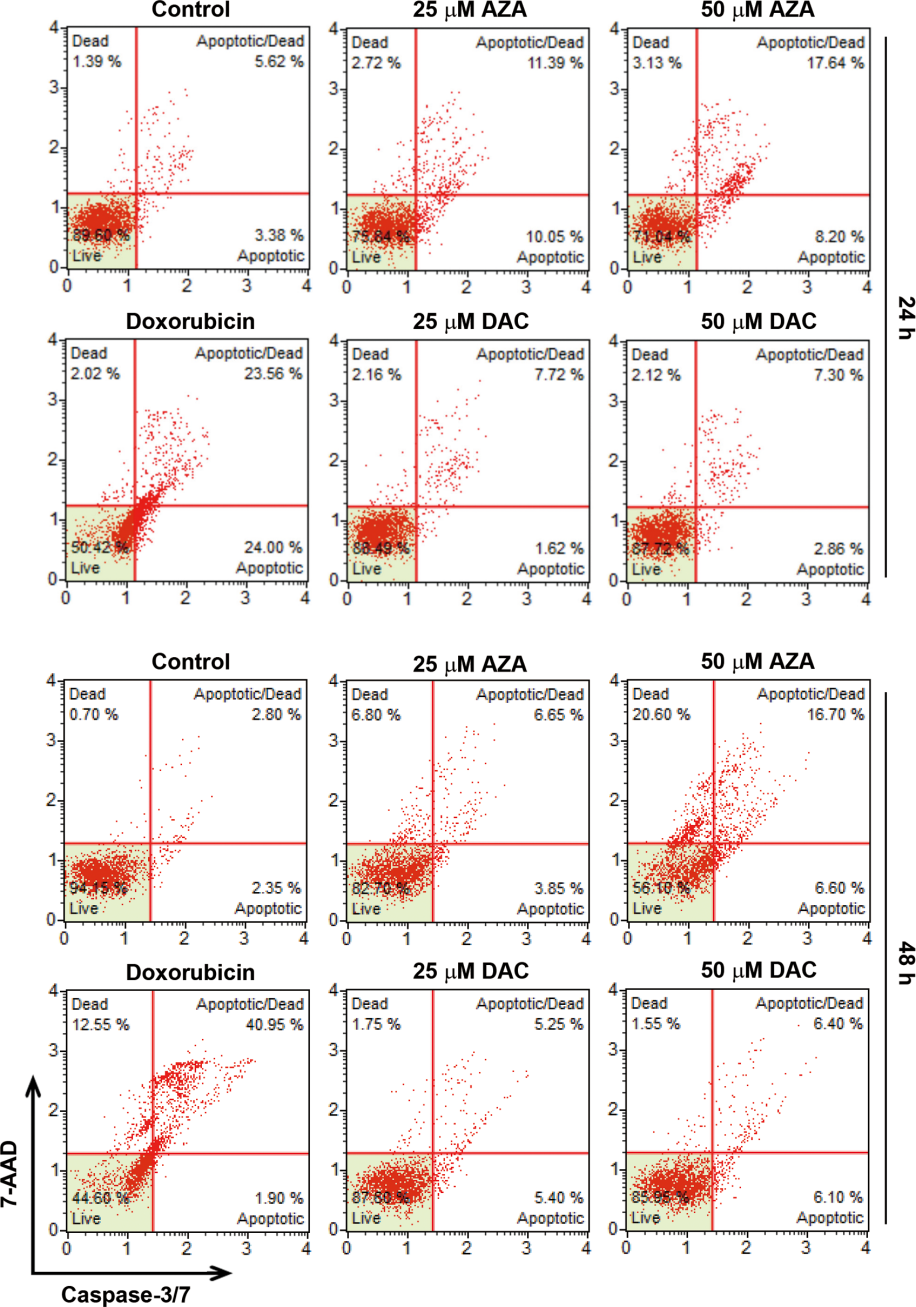
Effects of azacytidine (AZA) and decitabine (DAC) on caspase-3/7 activity HCT116 cells were treated with different doses of AZA or DAC, or 0.5 μM doxorubicin for 24 and 48 h, and the caspase-3/7 activity was analyzed by flow cytometry as described in “Materials and Methods”.

**Figure 7:**
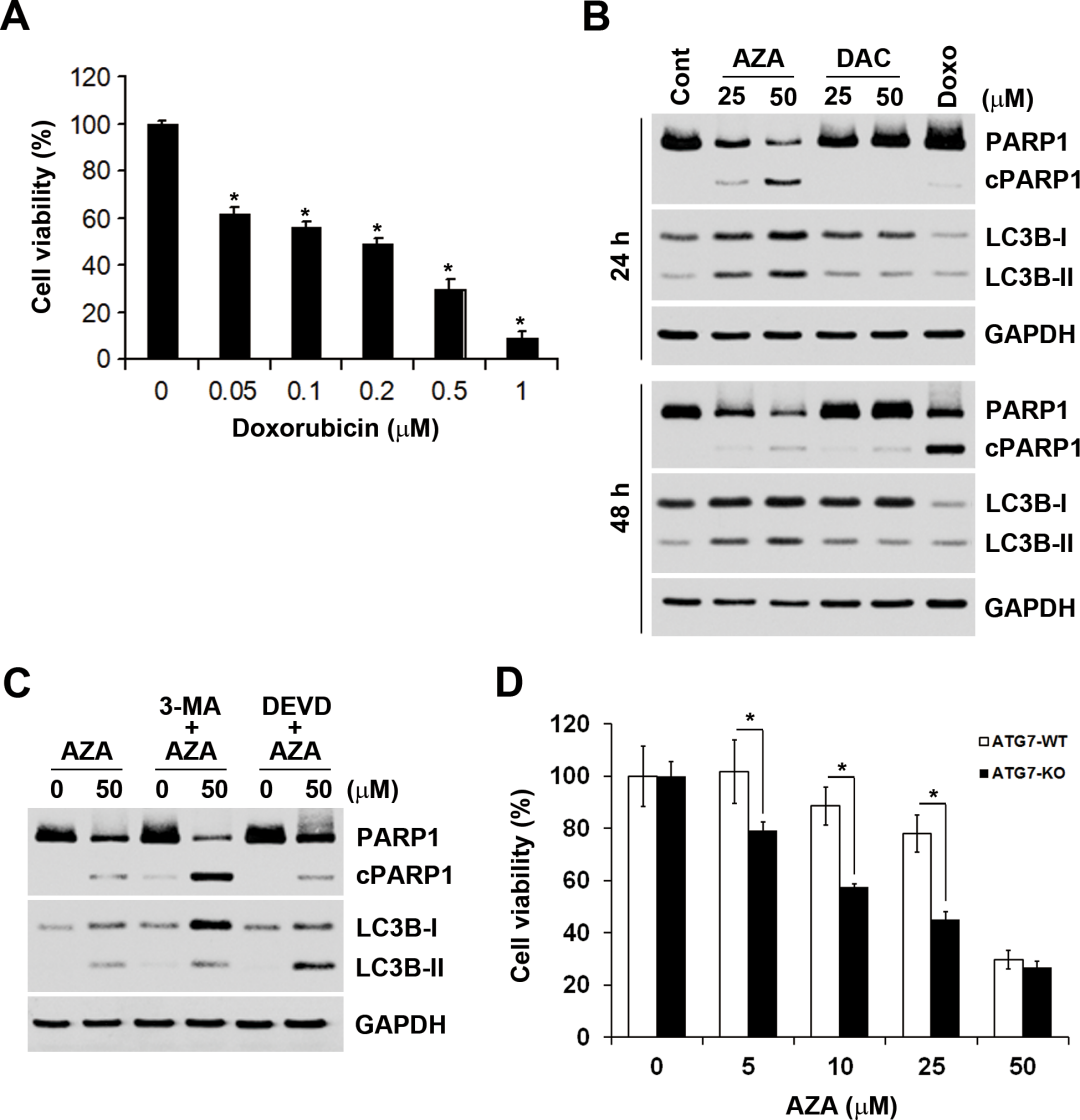
The relationship between apoptosis and autophagy induced by azacytidine (AZA) (**A**) HCT116 cells were treated with different doses of doxorubicin for 72 h, and the cell viability was analyzed by an MTT assay. (**B**) HCT116 cells were treated with different doses of AZA or decitabine (DAC), or 0.5 μM doxorubicin for 24 and 48 h, and whole-cell lysates were subjected to a Western blot analysis using antibodies against PARP1, LC3B, or GAPDH. (**C**) HCT116 cells were pretreated with 5 mM 3-MA or 50 μM Z-DEVD-FMK for 1 h and then exposed to 50 μM AZA for 24 h. Whole-cell lysates were subjected to a Western blot analysis using antibodies against PARP1, LC3B, or GAPDH. (**D**) ATG7-WT and ATG7-KO DLD-1 cells were treated with different doses of AZA for 72 h, and cell viability was analyzed by an MTT assay.

Induction of apoptosis by AZA might explain the differential effects of AZA and DAC on cell viability. Therefore, we further characterized AZA-induced apoptosis by a caspase-3/7 activity assay. In addition, cellular plasma membrane permeabilization was determined by staining of dead cells with the dye, 7-AAD. As shown in Figure 6, only AZA induced obvious activation of caspase-3/7 at 24 and 48 h. Treatment with 0.5 μM doxorubicin was used as a positive control for caspase-3/7 activation (Figure 6). Interestingly, we found that kinetic changes of AZA (50 μM) and doxorubicin (0.5 μM) on caspase activation differed, although their inhibitory effects on cell viability were similar (Figures 2C, 7A). The percentages of apoptotic, apoptotic/dead, and dead cells induced by doxorubicin (0.5 μM) at 24 h were 24%, 23.56% and 1.39%, respectively. At 48 h, the reduced apoptotic cell (1.9%) population was correlated with the induction of apoptotic/dead (40.95%) and dead (12.55%) cell populations (Figure 6), representing a shift from early to late apoptotic responses. However, AZA (50 μM) induced 8.2% of apoptotic, 17.64% of apoptotic/dead, and 3.13% of dead cells at 24 h, but 6.6% of apoptotic, 16.7% of apoptotic/dead, and 20.6% of dead cells at 48 h (Figure 6), indicating that the ability of AZA to induce apoptosis was limited as observed in a cell cycle analysis showing similar subG_1_ populations (18.1% and 19.1%, respectively) at 24 and 48 h (Figure 5A). Because poly(ADP ribose) polymerase 1 (PARP1) is a well-known cellular substrate of caspase-3/7 [27], the effects of AZA, DAC, and doxorubicin on PARP1 cleavage were examined by Western blot analyses. As shown in Figure 7B, AZA, but not DAC or doxorubicin, induced obvious cleavage of PARP1 after 24 h of treatment. Interestingly, the cleaved form of PARP1 declined without a concomitant recovery of the pro-form in response to 48 h of AZA treatment, suggesting that AZA-induced apoptosis might have been retarded. Consistent with results of caspase-3/7 staining (Figure 6), doxorubicin induced significant PARP1 cleavage at 48 h (Figure 7B). Therefore, these results suggest that AZA induced an unusual and acute apoptotic response compared to doxorubicin at an equivalent dose level which inhibited cell viability.

Autophagy is a physiological process that regulates the turnover of proteins and intracellular organelles. Through self-digestion, it provides an alternative energy source and serves as a temporary survival mechanism during starvation [28]. In addition, autophagy can be a stress adaptive method that avoids cell death in several scenarios, and it can also act as an antagonist to block apoptosis by promoting cell survival [29, 30]. Because of the inhibitory effect of AZA on protein synthesis, we hypothesized that AZA might simultaneously induce autophagy and apoptosis in a mutually exclusive manner. To investigate whether AZA induces autophagy in HCT116 cells, a Western blot analysis was performed to detect autophagy by the conversion of cytosolic LC3B-I (autophagy-inactive) to processed LC3B-II (autophagy-active) [31]. Indeed, AZA, but not DAC or doxorubicin, induced the conversion of LC3B-I to LC3B-II at both 24 and 48 h (Figure 7B). To investigate the role of autophagy in AZA-induced apoptosis, 3-methyladenine (3-MA), an autophagy inhibitor, was used. 3-MA is a class III PI3K inhibitor that blocks autophagosome formation in the early stage of autophagy [31]. As shown in Figure 7C, 3-MA blocked the conversion of LC3B-I to LC3B-II as indicated by an increase in LC3B-I, and concurrently enhanced AZA-induced PARP1 cleavage. In contrast, the caspase-3 inhibitor, Z-DEVD-FMK, inhibited AZA-induced PARP1 cleavage as indicated by an increase in the pro-form of PARP1, but enhanced AZA-induced LC3B-II accumulation (Figure 7C). To confirm the role of autophagy in the cytotoxicity of AZA, autophagy-deficient ATG7-knockout (ATG-KO) DLD-1 cells were used. As shown in Figure 7D, ATG7-KO DLD-1 cells were more sensitive to treatment with AZA. Therefore, AZA simultaneously induces apoptosis and cytoprotective autophagy in a mutually exclusive manner.

## DISCUSSION

It is now commonly accepted that polypharmacology (drug molecules that interact with multiple targets) is a basic property of small molecules, which is also an important basis for drug repurposing [2]. Therefore, investigating the polypharmacologic mechanisms of drugs would be very helpful for drug discovery. More and more biomedical databases and analytical tools have been developed in recent years, which provide easy and open access to the masses of accumulated data [3]. Integrating these resources would be highly useful in the field of polypharmacology. In this study, we demonstrated the value of an integrated chemical genomics approach that can be applied for drug repurposing and also for the discovery of novel mechanisms of unknown drugs.

Although AZA and DAC have been clinically used for more than half a century, and despite decades of research to define their mechanisms, little is known about their precise mechanisms of action [32]. In addition, despite being viewed as structurally and mechanistically similar drugs, AZA and DAC have different metabolism and destinations in cells, further increasing the complexity for delineating these two drugs. In this study, we proposed a workflow for an integrated chemical genomics approach, which indeed suggested functional disparities between AZA and DAC, because similar compounds and genes associated with AZA or DAC greatly differed, and AZA and DAC showed little similarity to each other. This is consistent with the highly distinct differences observed in microarray gene expression and proteomic profiles of AZA and DAC in cancer cells [15–18]. On further analyses of chemical and genetic profiles of AZA and DAC, we found that AZA inhibited protein synthesis and reduced protein stability, and then induced an acute apoptotic response and cytoprotective autophagy in a mutually exclusive manner. In contrast, DAC affected cell cycle progression and induced expressions of p53 and its target gene, p53R2. Therefore, these proof-of-concept studies indeed demonstrated the value of this systematic polypharmacological approach.

AZA and DAC are viewed as hypomethylating agents because they were shown to inhibit DNA methylation [33], which seems to be relevant to their clinical benefits [32]. However, their hypomethylating activity is lost and is replaced by direct cytotoxicity when higher concentrations are given [17], which is consistent with their original purposes of being developed as classical cytostatic agents [34]. In this study, differential effects of AZA and DAC were observed at doses of > 10 μM, suggesting that DNA-hypomethylating activities might be not associated with their functional disparities. However, it still cannot explain why the direct cytotoxicity of AZA and DAC toward cancer cells differed. Because 100% of DAC is incorporated into DNA, whereas 80%∼90% of AZA is incorporated into RNA and only 10%∼20% of it into DNA [10], RNA-dependent effects of AZA might have higher cytotoxicity than DNA-dependent effects of DAC. Incorporation of AZA into newly synthesized RNA (including ribosomal (r)RNAs, transfer (t)RNAs, mRNAs, and microRNAs) might interfere with their biogenesis and thus inhibit protein synthesis. Indeed, our results showed that the primary pathway influenced by AZA was aminoacyl-tRNA biosynthesis, and AZA was able to inhibit protein synthesis and stability. Incorporation of DAC into newly synthesized DNA might affect DNA replication that could cause arrest of the cell cycle and activation of the p53 signaling pathway to repair DNA damage [35]. Pathway enrichment of DAC-associated genes showed the involvement of cell cycle regulation and the p53 signaling pathway. In addition, DAC induced G_2_/M arrest and expressions of p53 and p53R2. p53R2 is a p53 target gene that causes G_2_/M arrest, and it is directly involved in the p53 checkpoint for repairing DNA damage and preventing cell death [26]. Therefore, unlike DAC, AZA did not induce p53 or p53R2 expression, and thus AZA-induced DNA damage could not be repaired.

In recent years, increasing numbers of studies have been conducted to investigate the RNA-dependent effects of AZA, and some specific molecular targets were identified. For example, RRM2, a subunit of ribonucleotide reductase, was identified as a novel molecular target of AZA in acute myeloid leukemia. The inhibition of RRM2 expression by AZA involves its direct RNA incorporation and an attenuation of RRM2 mRNA stability [11]. Because a reduction in the diphosphate form of AZA into deoxy-diphosphates by ribonucleotide reductase is required for the incorporation of AZA into RNA, inhibition of RRM2 can explain the small DNA-incorporating ratio of AZA. In addition, AZA was shown to inhibit tRNA methylation at DNMT2 target sites [36]. DNMT2 is a tRNA-specific methyltransferase with three verified tRNA targets: Asp-tRNA, Gly-tRNA, and Val-tRNA [37, 38]. It is of interest to note that the major pathway enriched in AZA-associated genes was aminoacyl-tRNA biosynthesis, and tRNA methylation by Dnmt2 promotes tRNA stability and protein synthesis in mice [39]. Therefore, we proposed that AZA treatment demethylated and destabilized tRNA, thus inhibiting protein synthesis.

Our results demonstrated that AZA induced an acute apoptotic response that had distinct kinetics compared to a chemotherapeutic agent, doxorubicin. According to this study, aminoacyl-tRNA might be the primary target of AZA. Disruption of aminoacyl-tRNA biosynthesis might be associated with AZA-induced apoptosis. One of the major apoptosis pathways that exists in mammalian cells is the mitochondrial pathway [40]. This pathway is defined by the release of mitochondrial cytochrome c into the cytosol where it binds to Apaf-1 and ATP/dATP, which then assembles into an oligomeric apoptosome complex. The apoptosome then recruits and oligomerizes the precursor of an initiator caspase, caspase-9, leading to its autoproteolytic activation. Caspase-9 activates effector caspases such as caspases-3 and -7, which cleave various cellular proteins, leading to cell death [40]. Interestingly, tRNA was shown to bind to cytochrome c, preventing its interaction with Apaf-1, thus blocking Apaf-1 oligomerization and caspase activation [41]. AZA might destabilize tRNA and then induce an acute apoptotic response in cancer cells, which warrants further investigation in the future.

In conclusion, we report how an integrative gene expression-based chemical genomics approach can be useful for identifying polypharmacological action mechanisms of a drug. Our results may provide novel molecular insights into the anticancer mechanisms of AZA.

## MATERIALS AND METHODS

### Library of integrated cellular signatures (LINCS) analysis

Data of the LINCS were generated by L1000 technology that only measures 1000 genes in each experiment, and the remaining about 22,000 genes are estimated by a model built from computational processing of thousands of gene expression datasets from the Gene Expression Omnibus (GEO) [13, 42, 43]. The LINCS has approximately one million gene expression profiles from 22,412 unique perturbations applied to 56 different human primary and cancer cell lines [43]. It not only provides chemical perturbations, but also genetic perturbations (knockdown and overexpression of a single gene). The LINCS can be queried by an input of the user's own up- and downregulated gene lists (gene symbol or Affymetrix U133A probe ID), or by using the “Compound Digest” or “Gene Digest” algorithm to search established drugs or genes. Outputs are lists of matching experiments in three tables of “Compound Connections”, “Consensus Knockdown Connections” and “Overexpression Connections”.

In this study, connections of AZA or DAC to other compounds or genes were directly obtained from the LINCS database (http://www.lincscloud.org/) using the “Compound Digest” algorithm. The output of results included “Compound Connections”, “Consensus Knockdown Connections”, and “Overexpression Connections”, representing the mostly similar (with positive scores) or dissimilar (with negative scores) compounds or genes, when they are knocked down or overexpressed, to AZA or DAC. The top 100 compounds with positive scores in “Compound Connections” are described in Supplementary Table S1, and the top 100 genes with positive scores in the “Consensus Knockdown Connections” and “Overexpression Connections” are described in Supplementary Table S2. “Score-best6” signifies the mean connectivity score across the six cell lines in which the pertubagen connected most strongly to the query. “ncell” signifies the number of cell lines over which the connectivity between the query and the perturbagen are summarized. “nsig” signifies the total number of perturbagen signatures over which its connectivity to the query is summarized. In this study, compounds with a Score-best6 of > 90 and genes with a rank of < 100 and/or a Score-best6 of > 60 were considered for further analyses.

### Search tool for interactions of chemicals (STITCH) analysis

LINCS compounds similar to AZA or DAC (with a Score-best6 of > 90) were queried with STITCH 4.0 (http://stitch.embl.de/) [6]. Compounds that are present in the STITCH database are described in Supplementary File S1. Different nominations between the LINCS and STITCH were carefully checked and manually edited. The organism in STITCH was set to “*Homo sapiens*”. To focus on compound connections and exclude the disturbance of proteins, the parameters were set as follows: Active prediction methods = Predictions; Required confidence (score) = medium confidence (0.400); and Interaction shown: no more than 0 interactors.

### Pathway enrichment analysis by the WEB-based GEne SeT AnaLysis Toolkit (WebGestalt)

Genes with a rank of < 100 and/or a Score-best6 of > 60 (Supplementary Table S2) were selected for a pathway enrichment analysis using the WebGestalt (http://bioinfo.vanderbilt.edu/webgestalt/) [14]. Because of limited genes in “Overexpression Connections” of AZA and DAC, only genes in the “Consensus Knockdown Connections” of AZA and DAC were considered for the WebGestalt analysis. Parameters were set as follows: Enrichment Analysis = KEGG Analysis; Select Reference Set for Enrichment Analysis = hsapiens_genome; Statistical Method = Hypergeometric; Multiple Test Adjustment = BH; Significance Level = 0.01; and Minimum Number of Genes for a Category = 5.

### Materials

RPMI-1640 medium, L-glutamine, sodium pyruvate, and Antibiotic-Antimycotic Solution (penicillin G, streptomycin, and amphotericin B) were purchased from Life Technologies (Gaithersburg, MD, USA). Fetal bovine serum (FBS) was purchased from Gibco (Grand Island, NY, USA). DNMT1, c-MYC, p53, p53R2, γH2AX, LC3B, and GAPDH antibodies were purchased form GeneTex (Hsinchu, Taiwan). The PARP1 antibody was purchased from Cell Signaling Technology (Beverly, MA, USA). The anti-puromycin antibody was purchased from Merck Millipore (Billerica, MA, USA). Horseradish peroxidase (HRP)-labeled goat anti-rabbit and anti-mouse secondary antibodies were purchased from Jackson ImmunoResearch (West Grove, PA, USA). 5-Azacytidine (AZA), 2′-deoxy-5-azacytidine (DAC), cycloheximide (CHX), and 3-methyladenine (3-MA) were purchased from Cayman Chemical (Ann Arbor, MI, USA). Doxorubicin was purchased from LC Laboratories (Woburn, MA, USA). Z-DEVD-FMK was purchased from R&D Systems (Minneapolis, MN, USA). 3-(4,5-Dimethylthiazol-2-yl)-2,5-diphenyl tetrazolium bromide (MTT), dimethyl sulfoxide (DMSO), puromycin, propidium iodide (PI), and ribonuclease A (RNase A) were purchased from Sigma (St. Louis, MO, USA). Protease and phosphatase inhibitor cocktails were purchased from Roche (Indianapolis, IN, USA). The ponceau S solution was purchased from SERVA (Heidelberg, Germany). Other chemicals or reagents not specified were purchased from OneStar Biotechnology (New Taipei City, Taiwan).

### Cell culture

Human colon cancer cells (HCT116, LoVo, RKO, HCT-15, DLD-1, and HT-29) were kindly provided by Prof. Ya-Wen Cheng (Taipei Medical University, Taipei, Taiwan). ATG7-wildtype (ATG7-WT) and ATG7-knockout (ATG7-KO) DLD-1 cells were purchased from Horizon Discovery (Cambridge, UK). These cells were cultured in RPMI-1640 medium supplemented with 10% FBS, 1 mM sodium pyruvate, 1% L-glutamine, and 1% Antibiotic:Antimycotic Solution, and incubated at 37°C in a humidified incubator containing 5% CO_2_.

### Cell viability assay

Cell viability was measured with an MTT assay. Cells were plated in 96-well plates and treated with drugs. After 72 h of incubation, 0.5 mg/mL of MTT was added to each well for an additional 4 h. The blue MTT formazan precipitate was then dissolved in 200 μL of DMSO. The absorbance at 550 nm was measured on a multiwell plate reader. IC_50_ values of AZA and DAC were calculated by SigmaPlot software using a standard curve analysis.

### Western blot analysis

Cells were lysed in an ice-cold buffer containing 50 mM Tris-HCl (pH 7.5), 150 mM NaCl, 1 mM MgCl_2_, 2 mM EDTA, 1% NP-40, 10% glycerol, 1 mM DTT, 1x protease inhibitor cocktail, and 1x phosphatase inhibitor cocktail at 4°C for 30 min. Cell lysates were separated on a sodium dodecylsulfate (SDS)-polyacrylamide gel, and then transferred electrophoretically onto a Hybond-C Extra nitrocellulose membrane (GE Healthcare, Piscataway, NJ, USA). The membrane was pre-hybridized in 20 mM Tris-HCl (pH 7.5), 150 mM NaCl, 0.05% Tween-20 (TBST buffer), and 5% skim milk for 1 h, and then transferred to a solution containing 1% bovine serum albumin (BSA)/TBST and a primary antibody and incubated overnight at 4°C. After washing with the TBST buffer, the membrane was submerged in 1% BSA/TBST containing an HRP-conjugated secondary antibody for 1 h. The membrane was washed with TBST buffer, and then developed with an enhanced chemiluminescence (ECL) system (Perkin-Elmer, Boston, MA, USA) and exposed to x-ray film (Roche).

### Puromycin-incorporation assay

Cells were plated in 6-cm dishes and treated with drugs for 6 and 24 h. One micromolar of puromycin was added 30 min before cells were harvested. Cell lysates were separated on a SDS-polyacrylamide gel, and then transferred electrophoretically onto a Hybond-C Extra nitrocellulose membrane. Proteins on the membrane were visualized by 0.2% ponceau S staining. The ponceau S stain was rinsed away, and the membrane was blotted with an anti-puromycin antibody.

### Flow cytometric analyses of the cell cycle and apoptosis

Cells were plated in 6-well plates for 24 h, and then treated with complete medium containing drugs for 24 and 48 h. Floating and adherent cells were harvested. For the cell cycle analysis, cells were immediately fixed with 75% ethanol and stored at −20°C. Cells were stained in staining buffer (10 μg/mL PI and 100 μg/mL RNase A) for 30 min and then analyzed on a Muse Cell Analyzer (Merck Millipore). For apoptosis determination, cells were stained with a Muse Caspase 3/7 Assay Kit (Merck Millipore) according to the manufacturer's instructions.

### Statistical analysis

Means and standard deviations of samples were calculated from the numerical data generated in this study. Data were analyzed using Student's *t*-test, and *p* values of < 0.05 were considered significant (*).

## SUPPLEMENTARY MATERIALS TABLES






