# Correction: Systematic discovery of drug action mechanisms by an integrated chemical genomics approach: identification of functional disparities between azacytidine and decitabine

**DOI:** 10.18632/oncotarget.26019

**Published:** 2018-08-17

**Authors:** Yao-Yu Hsieh, Tsui-Chin Huang, Hsiang-Ling Lo, Jyun-Yan Jhan, Shui-Tein Chen, Pei-Ming Yang

**Affiliations:** ^1^ PhD Program for Cancer Biology and Drug Discovery, College of Medical Science and Technology, Taipei Medical University and Academia Sinica, Taipei, Taiwan; ^2^ Division of Hematology and Oncology, Shuang Ho Hospital, Taipei Medical University, Taipei, Taiwan; ^3^ Graduate Institute of Cancer Biology and Drug Discovery, College of Medical Science and Technology, Taipei Medical University, Taipei, Taiwan; ^4^ Institute of Biological Chemistry, Academia Sinica, Taipei, Taiwan; ^5^ Department of Internal Medicine, Division of Hematology and Oncology, School of Medicine, College of Medicine, Taipei Medical University, Taipei, Taiwan

**This article has been corrected:** The author affiliation is given below:

**Yao-Yu Hsieh^1,2,5^**

^5^ Department of Internal Medicine, Division of Hematology and Oncology, School of Medicine, College of Medicine, Taipei Medical University, Taipei, Taiwan

Original article: Oncotarget. 2016; 7:27363-27378. https://doi.org/10.18632/oncotarget.8455

