# Supplementary material for: Systematic discovery of drug action mechanisms by an integrated chemical genomics approach: identification of functional disparities between azacytidine and decitabine
Source: Oncotarget. 2016 Mar 29;7(19):27363–78. doi: 10.18632/oncotarget.8455 (PMC5053656; doi:10.18632/oncotarget.8455)
Supplement: Supplementary file 1 [file oncotarget-07-27363-s001.pdf]

Systematic discovery of drug action mechanisms by an integrated chemical genomics approach: identification of functional disparities between azacytidine and decitabine

Supplementary Materials

Supplementary Table S1: The top 100 compounds similar to azacytidine (AZA) and decitabine (DAC)

Supplementary Table S2: The top 100 genes with gene expression profiles similar to AZA and DAC when they are knocked down or overexpressed
